# Arthroscopic inferior leaf meniscectomy of the involved anterior horn in the lateral meniscus horizontal tear via an accessary extreme far anteromedial portal

**DOI:** 10.1186/s12891-024-07384-3

**Published:** 2024-04-13

**Authors:** Na Guo, Cheng-bing Yang, An-hong Wang, Ying Jin, Shu-hong Wu, Hua-zhang Xiong

**Affiliations:** 1 Department of Orthopedic Surgery, Affiliated Hospital of Zunyi Medical University, 149# Dalian Road, Zunyi, 563003 People’s Republic of China; 2 Department of Orthopedic Surgery, People’s Hospital of Yinjiang Tujia and Miao Autonomous County, Yinjiang, 555200 People’s Republic of China

**Keywords:** Extreme far anteromedial portal, Efficacy, Lateral meniscus, Anterior horn, Horizontal tear, Meniscectomy

## Abstract

**Background:**

An accessory extreme far anteromedial portal can improve visualisation and ease inferior leaf meniscectomy in patients with lateral meniscal anterior horn horizontal tears. However, the therapeutic outcomes of adding an accessory extreme far anteromedial portal remain unclear. This study aimed to evaluate the clinical efficacy of adding an accessory extreme far anteromedial portal for treating lateral meniscal horizontal tears involving the anterior horns.

**Methods:**

This retrospective study included 101 patients with anterior horn involvement in lateral meniscal horizontal tears who underwent arthroscopic unstable inferior leaf meniscectomy between January 2016 and December 2020. The pathologies were diagnosed using physical examinations and magnetic resonance imaging. The anterior horn involved in the lateral meniscal horizontal tears was treated using inferior leaf meniscectomy. The primary endpoints were changes in the visual analogue scale, Lysholm, International Knee Documentation Committee, and Tegner scores at the final follow-up. The secondary endpoint was meniscal cure rate at 3 months postoperatively. The preoperative and postoperative functional scores were compared. The occurrence of complications was recorded.

**Results:**

All patients were followed up for an average of 4.9 ± 1.2 years (range 2.3–7.5 years). After 4 months, none of the patients experienced pain, weakness, instability, or tenderness in the lateral joint line, achieving an imaging cure rate of 98%. At the final follow-up, significant postoperative improvements were observed in the average values of the visual analogue scale score (3.5 ± 0.7 vs. 0.7 ± 0.6), Lysholm score (62.7 ± 4.4 vs. 91.8 ± 3.1), International Knee Documentation Committee score (61.9 ± 3.7 vs. 91.7 ± 9.5), and Tegner score (2.0 ± 0.7 vs. 6.1 ± 0.7). Excellent Lysholm scores were obtained in 81 patients, and good outcomes were obtained in 18 patients, with an excellent-to-good rate of 98.0%.

**Conclusions:**

Inferior leaf resection via the accessory far anteromedial portal is a safe treatment option for the involved anterior horn in lateral meniscal horizontal tears. This approach enhances visibility and facilitates surgical procedures, with minimal complications.

## Background

Tears of the lateral meniscus anterior horn (LMAH) are rare, with an incidence of less than 8%, identified through magnetic resonance imaging (MRI) and arthroscopic examinations [1, 2]. The outcomes of biomechanical studies have demonstrated that a torn meniscus can influence the stability and stress distribution of the knee [3–5], as well as the lubrication and nutrition of the cartilage [6]. Hence, arthroscopic partial meniscectomy is considered appropriate for treating irreparable meniscal injury [2]. Performing meniscectomy in the anatomic mode, as much as possible, promotes respect for joint biomechanics while preserving as much healthy tissue as possible [7].

Arthroscopic partial meniscectomy is an important technique for treating a horizontal tear of the lateral meniscus with excellent clinical outcomes [2, 8, 9]. This procedure is usually performed using the standardised anterolateral portal (ALP) and anteromedial portal (AMP), particularly in patients with mid-body and posterior horn tears of the lateral menisci. Although superior leaf or all-meniscectomy is easy for treating isolated LMAH tears using AMP and ALP, surgeons may encounter difficulty when using standardised portals for excising the inferior leaf for horizontal tears of the LMAH due to the limited visualisation and surgical space [10, 11].

To enhance visualisation and facilitate surgical procedure, several authors have proposed the use of a third portal. Chen et al. [6] used an inframeniscal portal to improve visualisation through AMP and facilitate unstable inferior leaf meniscectomy through inframeniscal portal. However, this approach has the disadvantage of potential synovial fistula formation after creating the lateral inframeniscal portal [10]. Kim et al. [12] introduced an accessory extreme far anteromedial portal (EFAMP) to improve visualisation and facilitate surgery. However, barring a few case reports and technique notes [11–13], no study has reported the therapeutic outcomes of inferior leaf meniscectomy via the standardised AMP and ALP combined with EFAMP approach in patients with horizontal tears of the LMAH.

The relationship between the clinical outcomes and surgery using the standardised AMP and ALP combined with EFAMP approach in patients with LMAH horizontal tears has not been clarified. Therefore, this retrospective study primarily aimed to evaluate the functional outcomes of patients with anterior horn involvement in lateral meniscal horizontal tears who underwent inferior leaf meniscectomy with the AMP and ALP combined with EFAMP approach. The secondary objective was to assess meniscal cure rates. We hypothesised that partial meniscectomy of the inferior leaf by adding an accessory EFAMP could improve functional outcomes and cure rate with a beneficial effect on enhancing visualisation and easing the surgical procedure.

## Methods

This study was approved by the ethics committee of the Affiliated Hospital of Zunyi Medical University (KLL-2023-560). All methods were performed in accordance with the Chinese Ethical Guidelines for Medical and Biological Research Involving Human Subjects and adhered to the principles outlined in the Declaration of Helsinki [14].

The information on surgical technique was extracted from the medical records of each patient who underwent an inferior leaf meniscectomy of the involved anterior horn in a lateral meniscus horizontal tear between January 2016 and December 2020. Surgeries of all patients included in this study were performed using a combination of AMP, ALP, and EFAMP by a senior surgeon with > 10 years of experience at our institution to minimise the influence of learning curve-related complications. The learning curve for the surgical method was completed according to a previously defined learning curve [15].

**Fig. 1:**
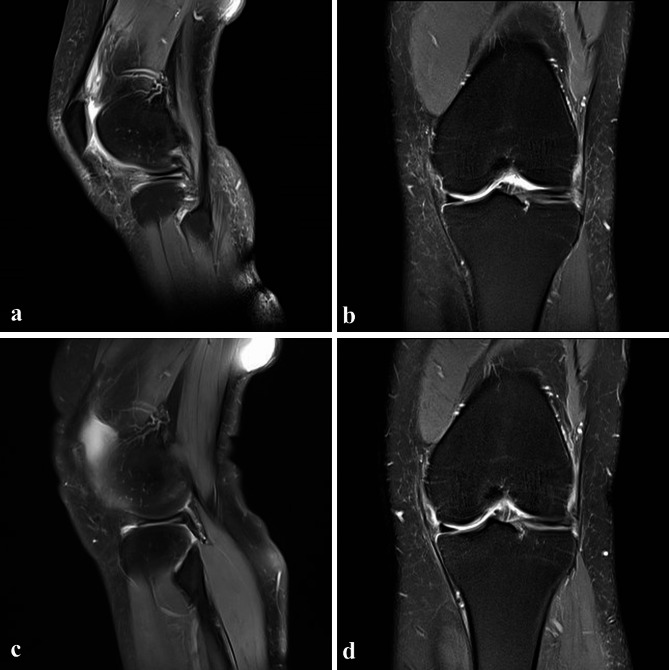
(**a** and **b**) Horizontal tear of the involved LMAH is observed in a sagittal and coronal MRI in a 26-year-old female patient. (**c** and **d**) Sagittal and coronal MRIs after 3 months of surgery reveal the resected inferior leaf of the LMAH. LMAH, lateral meniscus anterior horn; MRI: magnetic resonance imaging

The inclusion criteria were as follows: horizontal and irreparable involvement of the anterior horn in the lateral meniscus tear; inferior leaf meniscectomy using a combination of AMP, ALP, and EFAMP; presence of > 2 years follow-up records; American Society of Anesthesiologists (ASA) grade < 4; and age 14–55 years. We excluded patients with knee osteoarthritis (OA), as defined by Kellgren–Lawrence (KL) classification > 2 [8, 16], abnormal alignment of the involved lower limb (femorotibial angle (FTA) < 2.4° or > 7.2° observed on short knee radiographs; or hip-knee-ankle angle varus < 3° or valgus > 3° observed on full-length films) [17], previous knee surgery or traumas, septic or rheumatoid or gouty arthritis, a reparable red or red-white zone tear who underwent partial meniscectomy combined with suture or other procedures, severe articular cartilage injuries (> International Cartilage Repair Society grade 3) [8], severe ligament injuries, serious organic or infectious diseases, and no complete clinical data, along with those loss to follow-up. Each patient was diagnosed based on preoperative physical and imaging examinations. Physical examinations included assessment of joint line tenderness, knee joint effusion, and McMurray test. Imaging examinations encompassed standard weight-bearing anteroposterior (AP) and lateral radiographs, standing AP leg radiographs, and MRI scans. Standard weight-bearing AP and lateral radiographs were used to assess the presence of OA. Standing AP long-leg radiographs were used to evaluate lower limb alignment. All patients with horizontal and irreparable anterior horn involvement in a lateral meniscal tear were confirmed and diagnosed using MRI (Fig. 1a and b). The surgical indication was the involvement of anterior horn in the lateral meniscus horizontal tear with an unstable and irreparable inferior leaf of the white zone. All data were obtained and analysed retrospectively by reviewing medical records, MRI scans, and arthroscopic photographs.

### Surgical technique

All patients were administered general anaesthesia. All surgeries were performed with the patient placed in a supine position on an orthopaedic table. A tourniquet was applied around the thigh with an appropriate pressure based on blood pressure. Standardised ALP and AMP levels were established as described in a previous study [13]. The EFAMP is located 1 cm above the medial joint line, 3 cm medial to the margin of the patellar tendon, and nearly anterior to the medial edge of the medial femoral condyle [11–13]. The intraoperative status of the knee joint was examined using standard diagnostic arthroscopy. Once an involved anterior horn in the lateral meniscus horizontal tear was confirmed, the involved knee was positioned in a Fig. 4 configuration, and a probe hook was introduced to check the extent and configuration of horizontal tears thoroughly. The depth and extent of the cleft and the stability of the superior and inferior leaves were recorded. Partial meniscectomy of the unstable and irreparable inferior leaf was performed using a combination of AMP, ALP, and EFAMP, following a previously described technique [13]. Proper EFAMP site was palpated and localized with a finger depression. An 18-gauge spinal needle was inserted to pass the spot in front of the medial femoral condyle’s medial edge and approach transversely toward the LMAH to avoid damaging the articular cartilage.

**Fig. 2:**
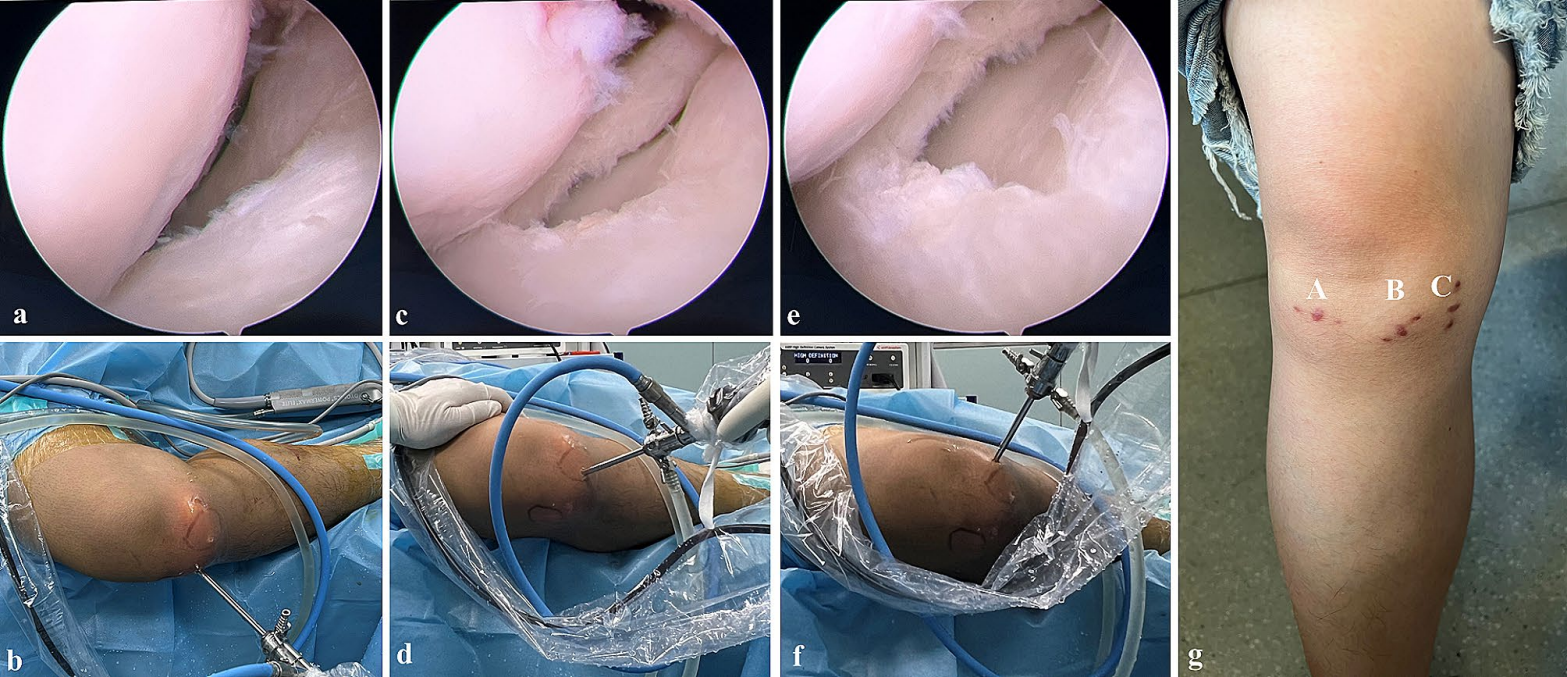
The visualisations are shown in the LMAH through ALP, AMP, and EFAMP, respectively. (**a** and **b**) A poor visualisation is observed in LMAH under arthroscopy through ALP. (**c** and **d**) A better visualisation is observed for LMAH under arthroscopy through AMP. (**e** and **f**) A best visualisation is observed in LMAH under arthroscopy through EFBMP. (**g**) ALP (**A**) AMP (**B**) and EFAMP (**C**) are located at the knee joint, respectively. ALP, anterolateral portal; AMP, anteromedial portal; EFAMP, extreme far anteromedial portal; LMAH, lateral meniscus anterior horn

The skin was incised using a 5 mm incision using a knife, a straight clamp was inserted along the spinal needle for blunt penetration into the soft tissue and capsule, and the portal was longitudinally expanded. After establishing the EFAMP, an arthroscope was introduced into the AMP to provide better visualisation of the LMAH. The superior leaf of the anterior horn was everted using a probe hook introduced through the ALP, and the inferior leaf was elevated and fixed. The inferior leaf of the LMAH was then excised using a left or right rotary basket clamp through the EFAMP. During the procedure, the surgeon intermittently discontinued the meniscectomy to determine the remnant inferior leaf under arthroscopy through EFAMP (Fig. 2a-g), and the surgical procedure was completed once the unstable inferior leaf was completely removed. A motorised shaver or radiofrequency was introduced to smoothen and contour the remaining rim of the meniscus. The incision was sutured, without applying any drainage tube.

### Postoperative management

All the patients were administered meloxicam for pain management. According to previous literature, exercises that strengthen the quadriceps, ankle pump exercises, and isometric contractions were started the day after surgery [18]. All patients were allowed to use their full range of motion in terms of knee mobility and flexion. Initiating ambulation was encouraged for enhancing the strength of the quadriceps to return to normal activities. The goal for hospital discharge was set at postoperative day 2. Follow-up was performed according to our institution’s standard postoperative schedule at 1, 2, 3, and 6 months and annually thereafter.

### Data Collection

After the complete data were collected from eligible patients, all patients with an involved anterior horn in the lateral meniscus horizontal tear were identified. The following data were collected: demographic characteristics, clinical and radiographic outcomes, and complications.

### Demographic and clinical characteristics

Patient data, including sex, age, body mass index (BMI), ASA grade, involved side, operative time, and follow-up time, were collected. The operative time was measured from the initiation of the skin incision to the completion of the incision suture.

### Clinical outcomes

The primary outcomes were functional scores, which were assessed by a senior author. Clinical cure was considered when there were no symptoms of meniscal tears, such as catching, locking, and giving way; no instability or weakness; and lateral knee-line tenderness. The functional outcomes were assessed preoperatively and at the final follow-up visit. The functional scores included the postoperative Lysholm score [19], International Knee Documentation Committee (IKDC) score [20], Tegner score [21], and visual analogue scale (VAS) score [22] by a senior author at each follow-up. The Lysholm score assesses knee function, with the sum of scores ranging from 0 (poorest) to 100 (highest). It is used to evaluate subjective symptoms; a total score of ≥ 90 is regarded as an excellent score, 84–90 as good, 65–83 as fair, and < 65 as poor [23]. The IKDC score assesses the ability and level to performance in various activities, with the highest numeric score of all questions representing the highest function. The Tegner score evaluates the highest current level of athletic participation, ranging from 0 (lowest) to 10 (highest). Knee pain was assessed using the VAS [22], which rates pain on a scale of 0 to 10 (0 = no pain and 10 = worst pain ever). Postoperative patient-reported outcomes were recorded and analysed to compare the differences between preoperative and postoperative outcomes.

### Radiographic evaluation

The secondary outcome was the meniscal cure rate, defined as the absence of tear signs on MRI after 3 months postoperatively. The outcomes of lower limb alignment and progression of knee OA were evaluated by two radiologists blinded to the surgeries and clinical outcomes. Lower limb alignment was evaluated using FTA on standing AP long-leg radiographs. FTA is defined as the lateral angle between the femoral and tibial axes [17]. A value of < 2.4° is considered varus, 2.4°–7.2° is considered neutral, and > 7.2° is considered valgus [17]. The progression of knee OA was evaluated using weight-bearing AP radiographs according to the KL classification. The KL classification assesses knee OA using grades from 0 (normal) to IV (most severe) [24]. Interobserver reliability was evaluated by applying interclass correlation coefficients (ICCs), which were interpreted using previously reported semi-quantitative criteria [25].

### Perioperative complications

We collected data on complications, including articular cartilage injury, medial collateral ligament (MCL) injury, muscular atrophy, incision-related conditions (oozing, delayed healing, and infection), knee stiffness, reoperation, and venous thromboembolism (VTE).

### Statistical analyses

The sample size for this study was calculated using Slovin’s formula, as described by Ellen [26]. It was determined according to the number of patients for the primary endpoint Lysholm score based on a two-sided 𝑡-test with a significance level of 5% reported by Chen et al. [6]. The required sample size was at least 32 patients for the Lysholm score with 80% power to detect a difference between the preoperative and postoperative Lysholm score. Statistical tests were performed using SPSS software SPSS® version 22 (SPSS Inc., Chicago, IL, USA) by a researcher blinded to surgical procedures and data collection. All values are expressed as the mean values with standard deviations. The Kolmogorov–Smirnov test was performed on each continuous variable to determine normality. The Student 𝑡-test was used for analysing continuous variables regarding the outcomes between the preoperative and postoperative. The chi-square or Fisher’s exact tests were used to compare categorical variables such as sex, age, and BMI. Interobserver reliability in measuring FTA and KL classification was evaluated using the ICC set at 95%. Values of *p* < 0.05 was considered significant.

## Results

### Patients

**Fig. 3:**
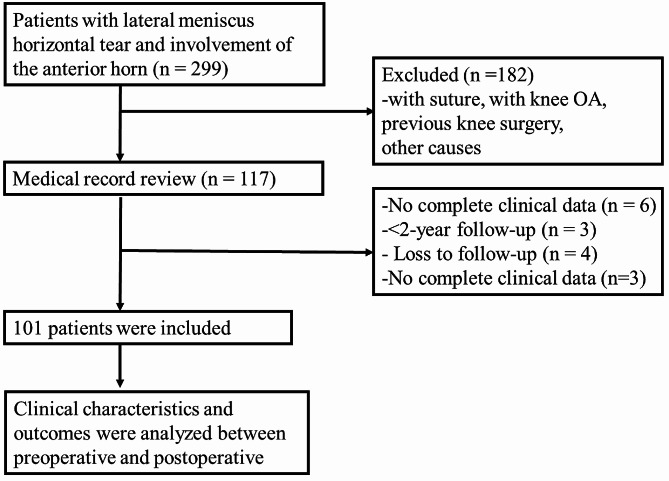
Flowchart of patient enrolment process. OA, asteoarthritis

Among the 299 screened patients, 198 were excluded from the study (Fig. 3). A retrospective review was conducted on data from 101 patients, spanning January 2016 to December 2020. Each patient required a minimum follow-up duration of 2 years (4.9 ± 1.2 years, range 2.3–7.5 years) for inclusion in the present study. In total, 55 patients had semilunar meniscus, 21 had mid-body and posterior horn horizontal tears, and the remaining had single-body tears. The shape of the other menisci was discoid, with 42 having horizontal tears in the mid-body and posterior horn, and the remaining cases being combined with isolated body tears. The sex, age, BMI, ASA grade, affected side, operative time, and follow-up duration of the patients are summarised in Table 1.

**Table 1:** Patient demographics and characteristics (*n* = 101)

Variable	Value
Age (years)	38.8 ± 9.2
**Sex**	
Male	64 (63.4%)
Female	37 (36.6%)
BMI (kg/m^2^)	24.6 ± 1.5
**ASA grade**	
I	73 (72.3%)
II	27 (26.7%)
III	1 (1%)
**Affected side (right/left)**	52/49
Right	52 (51.5%)
Light	49 (48.5%)
Operative time (Minutes)	32.5 ± 5.1
Follow-up time (years)	4.9 ± 1.2

ψ Continuous variables are expressed as the mean and standard deviation. Categorical variables were presented as numbers with percentages in parentheses. ASA, American Society of Anesthesiology; BMI, body mass index

### Clinical outcomes

At the final follow-up, no signs of tear, such as catching, locking, and giving way; no instability or weakness; and no lateral knee line tenderness were observed. The McMurray sign was negative in all the cases. Notably, the Lysholm, IKDC, Tegner, and VAS scores significantly improved postoperatively; with statistically significant differences from the preoperative values (*p* < 0.05) (Table 2). The excellent-to-good Lysholm score was 98.0% (Table 3).

**Table 2:** Clinical and radiographical outcomes of the patients (101 knees) with a follow-up of 4.9 ± 1.2 years ^ψ^

Variable	Preoperative value	Postoperative value	p-value
VAS score	3.5 ± 0.7	0.7 ± 0.6	< 0.001 ^a^
Lysholm score	62.7 ± 4.4	91.8 ± 3.1	< 0.001 ^a^
IKDC score	61.9 ± 3.7	91.7 ± 9.5	< 0.001 ^a^
Tegner score	2.0 ± 0.7	6.1 ± 0.7	< 0.001 ^a^
FTA (°)	5.9 ± 1.1	6.0 ± 1.0	> 0.05 ^a^

^ψ^Continuous variables are expressed as the mean and standard deviation

^a^ Independent-sample Mann–Whitney U test. FTA, femorotibial angle. IKDC, International Knee Documentation Committee; VAS, visual analogue scale

**Table 3:** The KL classification and mechanical alignment of the involved knee with a follow-up of 4.9 ± 1.2 years

Grade	0	I	II	III	IV
**KL classification**					
Preoperative values	86 (85.1%)	13 (12.9%)	2 (2.0%)	0	0
Postoperative values	81 (80.2%)	17 (16.8%)	3 (3.0%)	0	0
*p* value	0.643^a^				

Categorical variables are presented as numbers with percentages in parentheses

^a^ Fisher’s exact test. KL, Kellgren–Lawrence.

### Radiographic outcomes

At 3 months postoperatively, no signs of residual lateral meniscal tears in 99 knees were observed (Fig. 1c and d); in two cases, the torn inferior leaves were not fully removed. The radiographic cure rate was 98% at the final follow-up, and no significant differences in KL classification and FTA of the involved knee were observed (*p*˃0.05) (Tables 2 and 4). The ICCs for the preoperative evaluation of the KL classification and FTA were 0.938 and 0.960 and 0.946 and 0.935, respectively.

**Table 4:** Postoperative outcomes of Lysholm score grade and complications with a 4.9 ± 1.2 years follow-up

Variable	Value
Lysholm score grade	
Excellent	81 (80.2%)
Good	18 (17.8%)
Fair	2 (2%)
Poor	0
Excellent to good rate	99 (98.0%)
**Complications**	
MCL injury	1
Articular cartilage injury	0
Knee stiffness	0
Incision infection	0

Categorical variables are presented as numbers with percentages in parentheses

### Perioperative complications

At the latest follow-up, complications were observed in one patient with MCL relaxation (< grade I). No other complications, such as articular cartilage injury, knee stiffness, infection, neurovascular injury, or VTE were observed (Table 3).

## Discussion

This study revealed significant improvements in functional status and pain in patients who underwent arthroscopic unstable inferior leaf meniscectomy using the standardised AMP and ALP combined with EFAMP approach, yielding a remarkable cure rate of 98%. The standardised AMP and ALP combined with EFAMP approach is a reliable option for treating anterior horn involvement in lateral meniscal horizontal tears.

The application of the EFAMP was developed based on studies demonstrating surgical advantages of inferior leaf meniscectomy for LMAH in knee arthroscopy, which led to enhanced visualisation, ease of use, and reduced surgical difficulty [11–13]. Several case reports and technique notes have described the application of EFAMP in unstable inferior leaf meniscectomy for LMAH. In a case report by Park et al. [11], the application of EFAMP in inferior leaf meniscectomy for AHLM improved surgical visualisation and ease of performing surgery.

Inferior leaf meniscectomy for cleavage of the LMAH through the standardised AMP and ALP combined with EFAMP approach has been associated with improved arthroscopic visualisation, stabilisation, and resection of the inferior leaf. Despite the advantages of the standardized AMP and ALP combined with EFAMP approach, studies comparing the preoperative and postoperative clinical outcomes are lacking. The application of EFAMP’s clinical efficacy for unstable inferior leaf meniscectomy of the involved anterior horn in the lateral meniscus horizontal tear remains unclear. Our study addresses this knowledge gap and demonstrates significant improvements in clinical outcomes in pain and functional scores (VAS, Lysholm, IKDC, and Tegner scores), between the preoperative and postoperative periods, following the standardised AMP and ALP combined with EFAMP approach. Despite a 98% radiographic cure rate, the remaining patients showed no clinical signs or symptoms of meniscal tears. Furthermore, no significant differences in KL classification or FTA were observed between the preoperative period and the final follow-up. The significant functional recovery and good cure rate without OA progression suggest that the standardised AMP and ALP combined with EFAMP approach for inferior leaf meniscectomy of the involved anterior horn in lateral meniscal horizontal tears is safe and reliable.

In the present study, the standardised AMP and ALP combined with EFAMP approach posed a risk of intraoperative MCL injury in one patient, which could be related to the injury of the anterior bundle of the MCL while establishing EFAMP. The MCL, being wider and not palpable, may commonly cause partial MCL injury during EFAMP establishment. The MCL pie-crusting technique has been used to open up a tight medial compartment in patients with posterior root tears of the medial meniscus [27, 28]. Han et al. [28] conducted a study involving 60 patients with posterior root injury of the medial meniscus who underwent arthroscopic pie-crusting release of the posteromedial complex during meniscoplasty or meniscal suturing. They found that the pie-crusting release of the MCL can increase the posteromedial space and improve the visual field of the knee under arthroscopy, without causing residual valgus instability of the knee or affecting the clinical outcomes. Zhu et al. [27] reported treating 32 consecutive patients with posterior horn tears of the medial meniscus by pie-crusting MCL release at its tibial insertion using an 18-gauge intravenous needle. At the final follow-up, all patients exhibited negative results in the valgus stress test without instability and significant improvements in the clinical outcomes. These studies suggest that pie-crusting MCL release is a safe, minimally invasive, and effective surgical option. To reduce the risk of MCL injury, we made a 5 mm-length skin incision using a knife; a straight clamp was inserted along the spinal needle for blunt penetration into the soft tissue and capsule, and the portal was longitudinally expanded to reduce MCL injury. Hence, as described in the literature mentioned earlier, we believe that any potential minimal medial instability (grade 1 relaxation) during EFAMP establishment would be negligible. EFAMP can be safely and easily established without serious complications. Furthermore, it aids in approaching unstable inferior leaf meniscectomy of the involved anterior horn in lateral meniscal horizontal tears.

Adding EFAMP have several major advantages in preventing impingement of the meniscal anterior horn on surgical instruments, simplifying surgical technique, and improving visualisation. Establishing this portal under arthroscopic visualisation is quick, safe, and precise, requiring no special techniques or surgical instruments and minimising surgical difficulties. The surgical efficiency of EFAMP is superior to the inframeniscal portal, and the risk of occurring synovial fistula is eliminated [10]. When the inframeniscal portal is used for partial meniscectomy of the involved anterior horn in a lateral meniscus horizontal tear, it is technically difficult to completely observe the inferior leaf, which potentially leads to excessive or insufficient excision, damage to meniscus loop, and an increased risk of residual pain. Similar to the application of standard AMP and ALP portals alone, the surgical times would be extended. Although this study did not include any control group, the anterior horn involved in lateral meniscal horizontal tears was well treated with the addition of EFAMP. Similar results were obtained in previous studies [2, 8]. These outcomes align with our hypothesis that the standardised AMP and ALP combined with EFAMP approach would improve visualisation and ease surgical procedures for treating patients with an involved anterior horn in the lateral meniscus horizontal tear who underwent unstable inferior leaf meniscectomy.

This study has several unique attributes. First, we initially evaluated the clinical efficacy of the standard AMP and ALP combined with EFAMP approach for treating patients with an involved anterior horn in the lateral meniscus horizontal tear. Second, better functional status and pain reduction were achieved after surgery through the combined application of the three portals. Third, this study further confirmed that the standardised AMP and ALP combined with EFAMP approach could improve visualisation and ease of performing surgery with low complication rates.

The present study has several limitations. First, being a retrospective study without a control group, it inherently possesses limitations. However, the outcomes may have important implications for clinical practice and are encouraging. Second, the sample size was relatively small due to the low incidence of anterior horn involvement in lateral meniscus horizontal tears. Third, the style of the anterior horn involved in lateral meniscal horizontal tears was heterogeneous and variable. However, semilunar and discoid meniscal tears were well treated with the three-portal technique, validating the effectiveness of this new method for the involved anterior horn in lateral meniscus horizontal tears.

## Conclusions

Inferior leaf resection via accessory EFAMP is a safe option for treating involved anterior horn in lateral meniscal horizontal tears, demonstrating potential benefits in improving visualisation and facilitating surgical procedure without causing complications.

## Data Availability

The datasets generated and/or analysed during the current study are not public because we will enlarge the sample size and extend the follow-up time to further explore the relationship between clinical outcome and the AMP and ALP combined with EFAMP approach in unstable inferior leaf meniscectomy of patients with a horizontal tear of LMAH. However, these are available from the corresponding author upon reasonable request.

## Abbreviations

ALP: anterolateral portal
AMP: anteromedial portal
AP: anteroposterior
ASA: American Society of Anesthesiologists
BMI: body mass index
EFAMP: extreme far anteromedial portal
FTA: femorotibial angle
ICC: interclass correlation coefficients
IKDC: International Knee Documentation Committee
KL: Kellgren–Lawrence
LMAH: lateral meniscus anterior horn
OA: osteoarthritis
MCL: medial collateral ligament
MRI: magnetic resonance imaging
VAS: visual analogue scale
VTE: venous thromboembolism

## References

[1] Shepard MF, Hunter DM, Davies MR, Shapiro MS, Seeger LL (2002). The clinical significance of anterior horn meniscal tears diagnosed on magnetic resonance images. Am J Sports Med.

[2] Gan JZ, Lie DT, Lee WQ (2020). Clinical outcomes of meniscus repair and partial meniscectomy: does tear configuration matter?. J Orthop Surg (Hong Kong).

[3] Prince MR, Esquivel AO, Andre AM, Goitz HT (2014). Anterior horn lateral meniscus tear, repair, and meniscectomy. J Knee Surg.

[4] Shiwaku K, Kamiya T, Otsubo H, Suzuki T, Nabeki S, Yamakawa S (2022). Effect of anterior horn tears of the lateral meniscus on knee stability. Orthop J Sports Med.

[5] Koh JL, Zimmerman TA, Patel S, Ren Y, Xu D, Zhang LQ (2018). Tibiofemoral contact mechanics with horizontal cleavage tears and treatment of the lateral meniscus in the human knee: an in vitro cadaver study. Clin Orthop Relat Res.

[6] Chen D, Li Q, Sun Y, Qin J, Yao Y, Jiang Q (2017). Arthroscopic management for the unstable inferior leaf of the lateral meniscus anterior horn and associated cysts through a direct inframeniscal portal: a retrospective study. Biomed Res Int.

[7] Solarino G, Bortone I, Vicenti G, Bizzoca D, Coviello M, Maccagnano G (2021). Role of biomechanical assessment in rotator cuff tear repair: arthroscopic vs mini-open approach. World J Orthop.

[8] Lee SW, Chun YM, Choi CH, Kim SJ, Jung M, Han JW (2016). Single-leaf partial meniscectomy in extensive horizontal tears of the discoid lateral meniscus: does decreased peripheral meniscal thickness affect outcomes? (Mean four-year follow-up). Knee.

[9] Tsujii A, Matsuo T, Kinugasa K, Yonetani Y, Hamada M (2018). Arthroscopic minimum saucerization and inferior-leaf meniscectomy for a horizontal tear of a complete discoid lateral meniscus: report of two cases. Int J Surg Case Rep.

[10] Kim JM, Bin SI, Kim E (2009). Inframeniscal portal for horizontal tears of the meniscus. Arthroscopy.

[11] Park IH, Kim SJ, Choi DH, Lee SC, Park HY, Jung KA (2014). Meniscectomy of horizontal tears of the lateral meniscus anterior horn using the joystick technique. Knee.

[12] Kim SJ, Park IS (2004). Arthroscopic resection for the unstable inferior leaf of anterior horn in the horizontal tear of a lateral meniscus. Arthroscopy.

[13] Na SI, Woo MS, Lee JM, Kim MK (2013). A new surgical technique of arthroscopic partial meniscectomy for unstable inferior leaf of the anterior horn in a horizontal tear of lateral meniscus. Knee Surg Relat Res.

[14] Rits IA (1964). Declaration of Helsinki. Recommendations guidings doctors in Clinical Research. World Med J.

[15] Jackson WF, Khan T, Alvand A, Al-Ali S, Gill HS, Price AJ (2012). Learning and retaining simulated arthroscopic meniscal repair skills. J Bone Joint Surg Am.

[16] Kaminski R, Kulinski K, Kozar-Kaminska K, Wielgus M, Langner M, Wasko MK (2018). A prospective, randomized, double-blind, parallel-group, placebo-controlled study evaluating meniscal healing, clinical outcomes, and safety in patients undergoing meniscal repair of unstable, complete vertical meniscal tears (bucket handle) augmented with platelet-rich plasma. Biomed Res Int.

[17] Park A, Stambough JB, Nunley RM, Barrack RL, Nam D (2016). The inadequacy of short knee radiographs in evaluating coronal alignment after total knee arthroplasty. J Arthroplasty.

[18] Katz JN, Brophy RH, Chaisson CE, de Chaves L, Cole BJ, Dahm DL (2013). Surgery versus physical therapy for a meniscal tear and osteoarthritis. N Engl J Med.

[19] Briggs KK, Lysholm J, Tegner Y, Rodkey WG, Kocher MS, Steadman JR (2009). The reliability, validity, and responsiveness of the Lysholm score and Tegner activity scale for anterior cruciate ligament injuries of the knee: 25 years later. Am J Sports Med.

[20] Irrgang JJ, Anderson AF, Boland AL, Harner CD, Kurosaka M, Neyret P (2001). Development and validation of the international knee documentation committee subjective knee form. Am J Sports Med.

[21] Briggs KK, Steadman JR, Hay CJ, Hines SL (2009). Lysholm score and Tegner activity level in individuals with normal knees. Am J Sports Med.

[22] Ochiai S, Hagino T, Senga S, Saito M, Haro H (2014). Prospective evaluation of patients with anterior cruciate ligament reconstruction using a patient-based health-related survey: comparison of acute and chronic cases. Arch Orthop Trauma Surg.

[23] Tegner Y, Lysholm J. Rating systems in the evaluation of knee ligament injuries. Clin Orthop Relat Res. 1985:43–9. 4028566

[24] Kohn MD, Sassoon AA, Fernando ND (2016). Classifications in brief: Kellgren-Lawrence classification of osteoarthritis. Clin Orthop Relat Res.

[25] Munro BH (1997). Statistical methods for health care research.

[26] Ellen S. Slovin’s Formula Sampling Techniques. https://sciencing.com/slovins-formula-sampling-techniques-5475547.html (2020). Accessed December 5.

[27] Zhu W, Tang Q, Liao L, Li D, Yang Y, Chen Y (2017). [Application of pie-crusting the medial collateral ligament release in arthroscopic surgery for posterior horn of medial meniscus in knee joint]. Zhong Nan Da Xue Xue Bao Yi Xue Ban.

[28] Han X, Wang P, Yu J, Wang X, Tan H (2020). Arthroscopic pie-crusting release of the posteromedial complex of the knee for surgical treatment of medial meniscus injury. BMC Musculoskelet Disord.

